# Brain Freeze: Pembrolizumab-Induced Autoimmune Encephalitis in Triple-Negative Breast Cancer

**DOI:** 10.7759/cureus.74587

**Published:** 2024-11-27

**Authors:** Shivani K Modi, Charishma Bhimineni, Malay Rathod, Danielle Palaferro, Minal Dhamankar

**Affiliations:** 1 Internal Medicine, Einstein Medical Center Montgomery, East Norriton, USA; 2 Internal Medicine, Jefferson Einstein Montgomery Hospital, East Norriton, USA; 3 Medicine, Rutgers Monmouth Hospital, New Jersey, USA; 4 Hematology and Oncology, Albert Einstein Healthcare Network, Philadelphia, USA; 5 Hematology and Oncology, Einstein Medical Center Montgomery, East Norriton, USA

**Keywords:** expressive aphasia, immune-related adverse events, neurological complications, pembrolizumab, triple-negative breast cancer

## Abstract

This case report presents the first known instance of pembrolizumab-induced autoimmune encephalitis in a 41-year-old female patient with stage IIIc triple-negative breast cancer. The patient developed expressive aphasia three days after starting pembrolizumab in combination with chemotherapy, prompting comprehensive evaluations that ruled out infectious or metastatic causes. A diagnosis of pembrolizumab-associated autoimmune encephalitis was established following a lumbar puncture and MRI. After discontinuing the immune checkpoint inhibitors (ICI) and initiating high-dose corticosteroids (methylprednisolone), the patient's symptoms significantly improved within days. This case highlights the importance of recognizing and managing neurological immune-related adverse events (irAEs) associated with ICIs, as their incidence can be variable but potentially severe. Clinicians must maintain a high index of suspicion for neurological irAEs, especially with new neurological symptoms, as early diagnosis and intervention are crucial for optimizing patient outcomes while balancing effective cancer treatment. Ongoing research is essential to elucidate the mechanisms and management strategies for ICI-related autoimmune encephalitis.

## Introduction

Immune checkpoint inhibitors (ICIs) are a class of monoclonal antibodies that target regulatory immune checkpoint molecules that inhibit T-cell activation. By blocking co-inhibitory signaling pathways, ICIs enhance T cell-mediated anti-tumor immunity and promote immune-mediated tumor cell clearance [1]. Recognized for their antitumor effects, ICIs have been approved by the Food and Drug Administration (FDA) for the treatment of various malignancies, including melanoma, non-small cell lung cancer, colorectal cancer, and hepatocellular carcinoma. ICIs enhance the immune system's ability to recognize and attack tumors by blocking inhibitory pathways, such as CTLA-4 and PD-1/PD-L1, which suppress T-cell activity. However, by removing these "brakes," ICIs can inadvertently activate autoreactive T-cells that were previously dormant or suppressed, leading to immune-related adverse events (irAEs). These irAEs arise when the immune system mistakenly attacks healthy tissues, disrupting immune homeostasis and causing inflammation in organs such as the skin, liver, lungs, or muscles [1,2].

ICIs can be categorized based on the pathways they target, with monoclonal antibodies against CTLA-4 and those against PD-1/PD-L1 [2]. Among these, monoclonal antibodies against PD-1/PD-L1 are the preferred method for modern immunotherapy of solid tumors [2]. These ICIs enhance the anti-tumor immune response by lifting the inhibitory effect of PD-1 or PD-L1 on T-cell activation and proliferation and by reducing the number and/or inhibitory activity of Treg cells. This restoration of T-cell function leads to the inhibition of tumor development. However, by activating the immune system, ICIs can also nonspecifically disrupt immune homeostasis in non-tumor tissues, resulting in severe immune and inflammatory reactions known as immune-related adverse events (irAEs). The specific mechanisms underlying irAE development remain unclear, though they are generally believed to be linked to immune homeostasis disruption caused by ICI treatment [2]. ICs on the surface of non-tumor cells can bind with ICIs, leading to complement activation and increased inflammation, further disrupting immune homeostasis.

Common immune-related adverse events (irAEs) include skin rashes, pruritus, colitis, hepatitis, and various endocrine disorders. Nervous system-related irAEs are less frequent and can be divided into central nervous system (CNS) and peripheral nervous system (PNS) categories. PNS irAEs mainly consist of conditions like myasthenia gravis, Guillain-Barré syndrome, and peripheral sensory-motor neuropathy. CNS-related irAEs are much less common than PNS irAEs. Autoimmune encephalitis (AE) associated with CIs is a rare and poorly understood complication that has been infrequently reported [2,3].

## Case presentation

A 41-year-old female with a medical history of stage IIIc triple-negative breast cancer and hypertension presented to the emergency department with complaints of word-finding difficulty that had developed insidiously over the past three days. She was currently undergoing treatment with paclitaxel, pembrolizumab (Keytruda), and carboplatin, and had recently started on levatiracetam 500 mg BID for seizure management following a recent hospitalization. During her previous admission, MRI and CT scans showed no metastatic disease or significant abnormalities.

Upon presentation, the patient reported having difficulty expressing what she wanted to say, although she had no trouble understanding spoken language and could form the words mentally. She had no associated symptoms such as numbness, tingling, weakness, trauma, chest pain, shortness of breath, or signs of infection. The patient denied any seizure-like activity since her recent discharge. Her vital signs included a temperature of 37 °C, heart rate of 114 bpm, respiratory rate of 18 breaths/min, oxygen saturation of 98%, and blood pressure of 176/96 mmHg.

Initial evaluation in the ED revealed no acute abnormalities in her blood work. A CT scan of her head showed no new or acute findings, consistent with prior imaging. A neurological exam revealed expressive aphasia with intact naming, comprehension, and repetition but was otherwise unremarkable. The differential diagnosis included autoimmune encephalitis, leptomeningeal carcinomatosis, pembrolizumab-induced encephalitis, status epilepticus, and paraneoplastic encephalitis.

**Figure 1:**
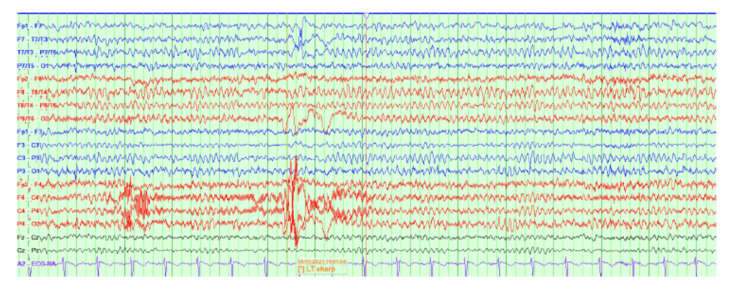
EEG showing focal left hemispheric cerebral dysfunction and a potential for seizures

An EEG indicated focal left hemispheric cerebral dysfunction and potential for seizures but did not record any events as shown in Figure 1. MRI of the brain revealed no acute intracranial abnormalities or pathologic enhancement; it showed nonspecific white matter signal abnormalities consistent with chronic migraine, early microvascular disease, demyelination, or infectious/inflammatory processes. A lumbar puncture was ordered for comprehensive analysis, including a polymerase chain reaction panel, cell count with differentials, oligoclonal bands, paraneoplastic evaluation, cryptococcal antigen, culture and gram stain, and JC Polyomavirus DNA. All the workup was negative, with cerebrospinal fluid (CSF) analysis showing WBC <5 as shown in Table 1.

**Table 1:** Lab work including CSF fluid with parameters seen in the patient

Parameters	Patient Values	Reference Range
White Blood Cell (WBC) Count	6.5 × 10³/µL	4.0–11.0 × 10³/µL
Hemoglobin	12.4 g/dL	12.0–15.5 g/dL
Platelet Count	320 × 10³/µL	150–400 × 10³/µL
Sodium	138 mEq/L	135–145 mEq/L
Potassium	4.2 mEq/L	3.5–5.0 mEq/L
Chloride	102 mEq/L	98–107 mEq/L
Bicarbonate	24 mEq/L	22–29 mEq/L
Blood Urea Nitrogen (BUN)	14 mg/dL	7–20 mg/dL
Creatinine	0.9 mg/dL	0.6–1.3 mg/dL
Glucose	112 mg/dL	70–99 mg/dL (fasting)
Calcium	9.1 mg/dL	8.5–10.2 mg/dL
Magnesium	2.0 mg/dL	1.7–2.2 mg/dL
Alanine Transaminase (ALT)	18 U/L	7–35 U/L
Aspartate Transaminase (AST)	21 U/L	8–33 U/L
C-Reactive Protein (CRP)	2.5 mg/L	<5.0 mg/L
Cerebrospinal Fluid (CSF): WBC	<5 cells/µL	<5 cells/µL
Cerebrospinal Fluid (CSF): RBC	0 cells/µL	0 cells/µL
Cerebrospinal Fluid (CSF): Glucose	65 mg/dL	40–70 mg/dL
Cerebrospinal Fluid (CSF): Protein	30 mg/dL	15–45 mg/dL

The treatment plan included a loading dose of levatiracetam 1.5 g, followed by 500 mg BID, and methylprednisolone 1g daily for five days. A proton pump inhibitor of 40 mg BID and sliding-scale insulin were initiated. Brain CTV was planned, and pembrolizumab was held due to the suspicion of an immune-related adverse event. 

An MRI of the brain revealed partially empty sella, scattered foci of T2 fluid-attenuated inversion recovery (FLAIR) hyperintensity in the cerebral white matter, and cerebellar tonsillar ectopia. These findings were nonspecific but consistent with chronic migraine, early microvascular disease, demyelination, or infectious/inflammatory etiologies. A CT scan of the brain showed mild periventricular white matter hypoattenuation, with no acute intracranial hemorrhage or mass effect, similar to previous scans.

The patient’s symptoms of expressive aphasia resolved within one or two days after starting methylprednisolone, and no further seizure events were noted. Final recommendations included awaiting CSF analysis results to confirm or rule out paraneoplastic encephalitis, infectious, or autoimmune causes. The patient was advised to complete the five-day course of Solu-Medrol and transition to prednisone 60 mg daily with a weekly taper. Pembrolizumab was discontinued due to grade 3 immune-related encephalopathy. Chemotherapy with weekly carboplatin and paclitaxel, followed by doxorubicin and cyclophosphamide, was to continue. The patient was scheduled for an outpatient follow-up with neurology and oncology for continued monitoring and treatment adjustments.

In conclusion, the patient’s clinical picture suggested an immune-related adverse event (IRAE) from pembrolizumab, leading to encephalitis. The immediate discontinuation of pembrolizumab and initiation of high-dose steroids resulted in rapid symptomatic improvement. Continued monitoring and adjustment of her chemotherapy regimen are necessary to balance effective cancer treatment and managing potential IRAEs.

## Discussion

This case highlights the intricate relationship between ICIs and the occurrence of irAEs, with a focus on pembrolizumab-induced autoimmune encephalitis. ICIs, including pembrolizumab, have significantly advanced the treatment of various malignancies by enhancing T-cell-mediated anti-tumor immunity. However, their capacity to nonspecifically activate the immune system can disrupt immune homeostasis, leading to irAEs. Immune checkpoint inhibitors (ICIs), such as pembrolizumab, work by blocking inhibitory signals like PD-1, enhancing T-cell-mediated anti-tumor immunity. However, this can disrupt immune tolerance, allowing autoreactive T-cells to become activated and attack normal tissues. In conditions like autoimmune encephalitis, this nonspecific immune activation results in inflammation and tissue damage in the brain, highlighting the potential for irAEs during ICI therapy [2].

Our patient, a 41-year-old female with stage IIIc triple-negative breast cancer, developed expressive aphasia shortly after initiating pembrolizumab, suggesting an immune-mediated central nervous system (CNS) irAE. Despite comprehensive initial evaluations, including MRI and CT scans, no metastatic disease or significant abnormalities were detected, supporting the diagnosis of an immune-mediated process. The patient’s rapid symptom resolution following high-dose steroids further supports the diagnosis of pembrolizumab-induced autoimmune encephalitis. Improvement upon discontinuing pembrolizumab and initiating immunosuppressive therapy underlines the importance of promptly recognizing and managing irAEs.

Autoimmune encephalitis is a rare but serious CNS irAE associated with ICIs. While the overall incidence of neurological irAEs is low, they are clinically significant, with recent studies indicating rates ranging from 3.2% to 12.0%, depending on the type of ICI and treatment regimen. The exact mechanisms underlying ICI-induced autoimmune encephalitis are not well understood but are thought to involve the activation of autoreactive T cells and subsequent CNS inflammation [3-5].

In this case, the absence of significant neuroimaging findings and the presence of nonspecific white matter changes highlight the challenges in diagnosing CNS irAEs. The scattered foci of T2 FLAIR hyperintensity in the cerebral white matter and cerebellar tonsillar ectopia were nonspecific but consistent with chronic migraine, early microvascular disease, demyelination, or infectious/inflammatory processes. This underscores the necessity for a high index of suspicion and a thorough diagnostic approach, including lumbar puncture and comprehensive cerebrospinal fluid (CSF) analysis, to exclude other potential causes and confirm the diagnosis.

Management of ICI-induced autoimmune encephalitis involves discontinuing the offending agent and initiating immunosuppressive therapy, typically high-dose corticosteroids. In our patient, a five-day course of Solu-Medrol followed by a tapering dose of prednisone led to rapid symptom improvement. This approach aligns with current guidelines for managing severe irAEs [2,3]. The decision to discontinue pembrolizumab was made considering the severity of the encephalitis and the need to balance effective cancer treatment with irAE management. While pembrolizumab has shown significant efficacy in treating triple-negative breast cancer, the risk of life-threatening irAEs requires careful monitoring and a multidisciplinary approach [6,7].

A review of the literature revealed 50 cases of ICI-associated autoimmune encephalitis, with patients’ ages ranging from 19 to 81 years and a mean age of 61.5 years. The male-to-female ratio was 1:1. The cancers involved included lung cancer, melanoma, renal cancer, pleural mesothelioma, Hodgkin’s lymphoma, and various other types. ICIs associated with these cases included nivolumab, ipilimumab, pembrolizumab, sintilimab, durvalumab, dostarlimab, and atezolizumab. The time from ICI initiation to AE symptom onset ranged from 4 days to 18 months, with a median of 3 months. CSF analysis commonly showed increased cell counts, elevated protein levels, and positive oligoclonal bands. Autoimmune antibodies in CSF or serum were present in 28 patients. The treatment outcomes varied, with 31 patients showing improvement after steroids, intravenous immunoglobulin (IVIg), plasma exchange, and rituximab, while 13 did not improve and 6 died. The mechanisms of ICI-associated autoimmune encephalitis may include cross-reactivity with CNS autoantigens, simultaneous enhancement of tumor and CNS immune responses, and direct neuronal damage by activated immune cells [3].

In addition to autoimmune encephalitis, ICIs can lead to other neurological complications, including peripheral neuropathies like myasthenia gravis, Guillain-Barre syndrome, chronic polyneuropathies, and mononeuropathies, as well as CNS complications such as noninfectious encephalitis, demyelinating disease, and cerebral artery vasculitis. The classification of irAEs into five grades helps guide treatment decisions, with Grade 1 irAEs allowing continued ICI use and Grades 2-4 requiring stronger immunosuppressive therapy and potential ICI discontinuation [8].

The treatment of ICI-associated autoimmune encephalitis should be individualized and cautious. IVIg or plasma exchange may be considered first-line treatments for Grade 2 and Grade 3 patients, with ICI discontinuation only if initial treatments are ineffective. Close monitoring is essential to balance effective cancer treatment and irAE management.

Learning points

Early recognition and prompt treatment of neurological irAEs are crucial to preventing severe complications. It is essential to develop treatment plans that effectively balance cancer therapy with the management of irAEs to ensure optimal patient outcomes. Ongoing research is necessary to gain a deeper understanding of ICI-induced encephalitis, as the mechanisms behind this rare complication remain poorly understood. High clinical vigilance, along with thorough diagnostics, are critical to accurately diagnose and address these rare but potentially serious side effects.

## Conclusions

This case illustrates the complexity and seriousness of immune-related adverse events (irAEs) due to immune checkpoint inhibitors (ICIs) such as pembrolizumab. Our patient, with stage IIIc triple-negative breast cancer, developed autoimmune encephalitis, presenting as expressive aphasia. Early recognition of neurological symptoms, prompt treatment with high-dose steroids, and a multidisciplinary approach involving oncology and neurology led to her rapid improvement. Managing irAEs requires personalized treatment plans that balance effective cancer therapy with the need to address adverse events. Ongoing research and increased awareness are crucial to understanding and managing these complications better. Clinicians should be vigilant for irAEs and perform comprehensive diagnostic workups when necessary.
